# *transparent*, a gene affecting stripe formation in Zebrafish, encodes the mitochondrial protein Mpv17 that is required for iridophore survival

**DOI:** 10.1242/bio.20135132

**Published:** 2013-06-03

**Authors:** Jana Krauss, Pantilis Astrinides, Hans Georg Frohnhöfer, Brigitte Walderich, Christiane Nüsslein-Volhard

**Affiliations:** Max-Planck-Institut für Entwicklungsbiologie, Spemannstrasse 35, 72076 Tübingen, Germany; * Present address: Universität Tübingen, Interfakultäres Institut für Biochemie, Hoppe-Seyler-Strasse 4, 72076 Tübingen, Germany

**Keywords:** Mpv17, Mitochondria, Chimeras, Iridophore

## Abstract

In the skin of adult zebrafish, three pigment cell types arrange into alternating horizontal stripes, melanophores in dark stripes, xanthophores in light interstripes and iridophores in both stripes and interstripes. The analysis of mutants and regeneration studies revealed that this pattern depends on interactions between melanophores and xanthophores; however, the role of iridophores in this process is less understood. We describe the adult viable and fertile mutant *transparent (tra)*, which shows a loss or strong reduction of iridophores throughout larval and adult stages. In addition, in adults only the number of melanophores is strongly reduced, and stripes break up into spots. Stripes in the fins are normal. By cell transplantations we show that *tra* acts cell-autonomously in iridophores, whereas the reduction in melanophores in the body occurs secondarily as a consequence of iridophore loss. We conclude that differentiated iridophores are required for the accumulation and maintenance of melanophores during pigment pattern formation. The *tra* mutant phenotype is caused by a small deletion in *mpv17*, an ubiquituously expressed gene whose protein product, like its mammalian and yeast homologs, localizes to mitochondria. Iridophore death might be the result of mitochondrial dysfunction, consistent with the mitochondrial DNA depletion syndrome observed in mammalian *mpv17* mutants. The specificity of the *tra* phenotype is most likely due to redundancy after gene multiplication, making this mutant a valuable model to understand the molecular function of Mpv17 in mitochondria.

## Introduction

Pigmentation is widespread among the animal kingdom. Functions of pigmentation range from vision, mimicry and environmental adaptation to UV-light protection and kin recognition. Teleosts evolved an impressive diversity in body colour patterns by varying the arrangement of pigmented cells (chromatophores) in the skin, producing different colours ranging from white and silvery over yellow to red, brown and black. Depending on the arrangement and combination of these chromatophores, fish can even produce green or blue colours in the skin (Kelsh, 2004).

The zebrafish *Danio rerio* owes its name to the regular pattern of horizontal dark blue stripes covering the flanks of the adult animal. This pattern is composed of three chromatophore types, black melanophores, yellow xanthophores and reflective iridophores. During recent years many mutants were collected that display an altered pigment pattern in adults, resulting either from a lack or reduction of a particular chromatophore type, or from alterations in the arrangement of pigment cells in the skin (Haffter et al., 1996; Kelsh et al., 1996; Odenthal et al., 1996; Parichy, 2003; Parichy, 2007; Rawls et al., 2001).

Body pigment cells in vertebrates derive from the neural crest, a transient highly migratory embryonic tissue (Le Douarin and Dupin, 2003). In zebrafish the larval pattern forms from chromatophores directly derived from the neural crest. Melanophores distribute in four characteristic stripes, which form a lateral stripe along the horizontal myoseptum, and stripes at dorsal and ventral positions of the myotome and along ventral positions of the yolk. Iridophores intersperse between melanophores except in the lateral stripe. Xanthophores cover the flanks of the larval trunk (Eisen and Weston, 1993; Kelsh et al., 1996; Raible and Eisen, 1994). During neural crest migration, in early embryogenesis, pigment stem cells are laid down, serving the development of the adult pattern that emerges during maturation of juvenile fish, a process called metamorphosis (Parichy, 2003). Adult melanophores arise from a small number of stem cells, which are established close to the ganglia of the peripheral nervous system (Dooley et al., 2013). Their progeny later migrate out along axons of the peripheral nervous system (Budi et al., 2008; Budi et al., 2011; Hultman et al., 2009; Hultman and Johnson, 2010). While populating the skin, melanophores develop the characteristic dark stripes in flanks, and caudal and anal fins. Yellow xanthophores and reflective iridophores populate the spaces between the dark stripes, the so-called interstripe regions of the adult flanks (Hirata et al., 2003; Hirata et al., 2005; Johnson et al., 1995; Kirschbaum, 1975; Maderspacher and Nüsslein-Volhard, 2003; Parichy et al., 2009).

While there is considerable knowledge about melanophores, especially regarding their specification, differentiation and melanin synthesis, the ontogeny of xanthophores and iridophores is less well understood. Iridophores produce large amounts of crystals mainly consisting of guanine, located subcellularly in iridosomes (“reflecting platelets”) (Hirata et al., 2005). Depending on the angle of light incidence, the reflections of the guanine crystals appear silvery to golden. Several mutants displaying impaired development of iridophores have been described. Mutations in *shady (shd)*, a gene coding for leucocyte tyrosine kinase (Ltk), cause a failure in iridophore specification and in consequence the lack of differentiated iridophores throughout life (Haffter et al., 1996; Kelsh et al., 1996; Lopes et al., 2008). *shd/ltk* was shown to be required cell autonomously in iridophores in larvae (Lopes et al., 2008). Mutations in another gene, *rose (rse)*, which encodes the endothelin receptor b1a (Ednrb1a), lead to an adult-specific reduction of iridophores (Johnson et al., 1995; Parichy et al., 2000a). Interestingly, both mutants, besides the defects in iridophores, show a strong reduction in melanophore numbers in adults (Haffter et al., 1996; Johnson et al., 1995; Lopes et al., 2008; Parichy et al., 2000a). One striking similarity of these mutants is the normal pigment pattern of caudal and anal fins, which led to the conclusion, that iridophores might play, if any, only a minor role in body stripe formation (Maderspacher and Nüsslein-Volhard, 2003; Parichy, 2003; Nakamasu et al., 2009). *shd/ltk* and *rse*/*ednrb1a* were shown to act cell autonomously in iridophores in adults, indicating that iridophores and their interaction with other chromatophores are required for stripe formation in the body (Frohnhöfer et al., 2013).

In another group of mutants iridoblasts are specified, however, pigment synthesis is compromised due to dysfunctional biosynthesis of guanosine-derivatives, which serve as intermediates in the synthesis of guanine. The affected genes encode Paics and Gart, two enzyme complexes involved in the synthesis of inosine monophosphate (IMP), a precursor of the purine nucleotides adenosine monophosphate (AMP) and guanosine monophosphate (GMP). Gart encodes phosphoribosylglycinamide formyltransferase, phosphoribosylglycinamide synthetase, phosphoribosylaminoimidazole synthetase, a trifunctional enzyme that catalyzes steps 2, 3 and 5 of inosinemonophosphate (IMP) synthesis. Paics encodes phosphoribosylaminoimidazole carboxylase, phosphoribosylaminoimidazole succinocarboxamide synthetase.

Furthermore, knockdown of guanosine monophosphate synthase (*gmps*) results in reduced iridophore and xanthophore pigmentation in larvae without obvious decrease in cell number (Ng et al., 2009). The expression of purine nucleoside phosphorylase 4a (pnp4a), whose product metabolizes guanosine to guanine, is highly upregulated in iridoblasts and iridophores and serves as an excellent marker for this cell type (Lang et al., 2009).

Here we present the molecular and phenotypic characterization of *transparent (tra)* (Walker and Streisinger, 1983). This mutant shows a strong reduction in iridophore pigmentation throughout life and additionally a reduction in melanophore numbers in adults. This renders the fish transparent, such that the inner organs can be observed through the skin. We show that the defect lies in a loss of iridophores. *tra* acts autonomously in iridophores, while mutant melanophores are able to contribute normally to stripes when confronted with wild type iridophores in chimeric animals. The mutant phenotype is due to a small deletion in *mpv17*, a human disease gene encoding a protein of the inner mitochondrial matrix. This suggests that mitochondrial function is crucial for the survival of this cell type. The melanophore stripe phenotype is secondary, caused by the lack of iridophores, indicating a dependence of melanophore development and patterning on the presence of iridophores.

## Materials and Methods

### Fish husbandry

We used fish of the following genotypes: *transparent^b6^*, *nacre^w2^*, *pfeffer^tm236b^*, *rse^tLF802^*, Tuebingen, WIK, *albino^b4^*. Zebrafish were maintained as described previously (Brand et al., 2002). Embryos and larvae were staged as described previously (Kimmel et al., 1995).

### Transplantation

Chimeric animals were generated by transplantation of 10–30 blastula cells into hosts of the same stage as described previously (Kane and Kishimoto, 2002). Animals were raised to adulthood and analysed for striped patches (“clones”). Typically, these covered large contiguous regions often spanning the entire flank from dorsal to ventral.

### Genetic mapping

Fish homozygous for *tra* were crossed to WIK. Their progeny was raised and incrossed and progeny was phenotyped for *tra*. Genetic linkage to linkage group 20 was determined as described previously (Geisler et al., 2007). Further fine mapping was achieved by the use of published microsatellite markers or SNPs. Candidate genes were chosen based on genome annotation (ensemble Zv9). For validation of candidate genes, genomic DNA for mapping was used or total RNA was prepared from both wild type and mutant embryos using TRIzol Reagent (Invitrogen) according to the manufacturer's protocol. Reverse transcription was performed using total RNA, oligo(dT) primer and Omniscript RT kit (Qiagen). Primers for amplification and sequencing of transcripts or genomic fragments were as follows:

slc30a2 forward: 5′-GAAATGGCTGGTGCTATTG

slc30a2 reverse: 5′-CTCACCGGTGTGCTGCAG

uckl1 forward: 5′-AAGCGTACTTCCGGACATG

uckl1 reverse: 5′-GCCTGCATTGAATGTGTGTAG

mpv17 forward: 5′-CGAGCATCACCTTATGTTG

mpv17 reverse: 5′-CTCGTGTTACATGTAATG

uts1 forward: 5′-GTCTTCTTCAGTGGCAGC

uts1 reverse: 5′-GAGTGTGAATGTCGGATG

trim54 forward: 5′-GATGAAGACTGGCACTGC

trim54 reverse: 5′-CCTCTTACTGAACTCAGAG

dnajc5ga forward: 5′-CTGACTCCTCTATGATTC

dnajc5ga reverse: 5′-CAGCGATAAGCACACTAC

### Apoptosis assays

TUNEL test: 24, 48, 72 and 96 hpf embryos and larvae were fixed in 4% PFA overnight at 4°C, three times washed in PBST (PBS containing 0.1% Tween-20) and treated with Proteinase K (5 µg/ml) (48 hpf for 10 min, 72 hpf for 20 min and 96 hpf for 35 min). After washing three times in PBST, apoptotic cells were detected using the *In Situ* Cell Death Detection Kit, TMR red (Roche) according to the manufacturer's protocol.

Acridine Orange test: 24 hpf, 48 hpf, 72 hpf and 96 hpf embryos and larvae were stained with Acridine Orange as described previously (Furutani-Seiki et al., 1996).

### RNA rescue

Mpv17 coding sequence was amplified with the primers mpv17 (see above) and subcloned into pCRII-TOPO (Invitrogen) followed by subcloning into pCS2+ using BamHI and PspOMI (NEB) and sequencing. For the mpv17:EGFP fusion construct, EGFP and mpv17 coding sequences were amplified (egfp oligos: 5′- GCT AGC GTG AGC AAG GGC GAG GAG and 5′- CTC GAG TTA CTT GTA CAG CTC GTC C; mpv17 oligos: 5′- GGA TCC CGA GCA TCA CCT TAT GTT G and 5′- GCT AGC CAT CTT GTT GGC TTT CCA G) and ligated into pCS2+ followed by sequencing. Both plasmids were linearized with NotI and subjected to *in vitro* transcription for synthesis of capped mRNA by mMESSAGE mMACHINE SP6 kit (Ambion). 0.2 ng RNA was injected into *tra* and wild type one-cell stage embryos according to standard methods. 5 dpf larvae were analyzed for iridophore characteristic silvery pigmentation.

### RNA *in situ* hybridization

Riboprobes for in situ hybridization were generated by an RT-PCR-based approach. Templates for RNA probes were amplified using an antisense DNA oligo containing a T7 promoter sequence on its 5′ end. The following DNA oligo combinations were used:

mpv17: 5′- GAC TGG CCA ATC ACA ACG and 5′- TGG ATC CTA ATA CGA CTC ACT ATA GGG TTC CAG ACA ACA GCC AC

pnp4a: 5′- GTT GGC GGA TGG GCT CAA G and 5′- TGG ATC CTA ATA CGA CTC ACT ATA GGG CAT TGA GAG GAT TCA GTC C

ednrb1: 5′- GCA CTG TGC TGG CTT CCA C and 5′- TGG ATC CTA ATA CGA CTC ACT ATA GGG GCT GTT ATG GCT GAT CCT C

These amplicons were subjected to DIG-labelled (DIG-UTP, Roche) *in vitro* transcription by T7 MEGAscript kit (Ambion). RNA *in situ* hybridization was carried out according to standard procedures. 20 embryos or larvae of wild type or *tra* were analysed.

### Electron microscopy

For transmission electron microscopy (TEM), 5 dpf larvae were fixed in 4% formaldehyde and 2.5% glutaraldehyde in PBS (pH 7.2) at 4°C overnight. Samples were postfixed with 1% osmium tetroxide, rinsed with water and block stained with 1% uranyl acetate for 1 h on ice, followed by dehydration in a graded ethanol series and infiltration with Epon. Samples polymerized at 60°C for 48 h. Ultra-thin sections were stained with uranyl acetate and lead citrate and viewed in a Philips CM10 electron microscope at 60 kV.

### Image aquisition

Adult fish were briefly anaesthetized with 0.004% MS-222 (Sigma) and imaged with Canon D5MarkII/MACRO 100. The angle of illumination had to be adjusted individually to allow optimal visualization of iridophore pigmentation. Images were taken on a 510 Meta confocal microscope (Zeiss), Axiophot fluorescence microscope (Zeiss) and Discovery stereo microscope (Zeiss). Photographs were processed in Adobe Photoshop.

## Results

### *tra* mutants display a reduced iridophore pigmentation in all stages of development

**Fig. 1.**
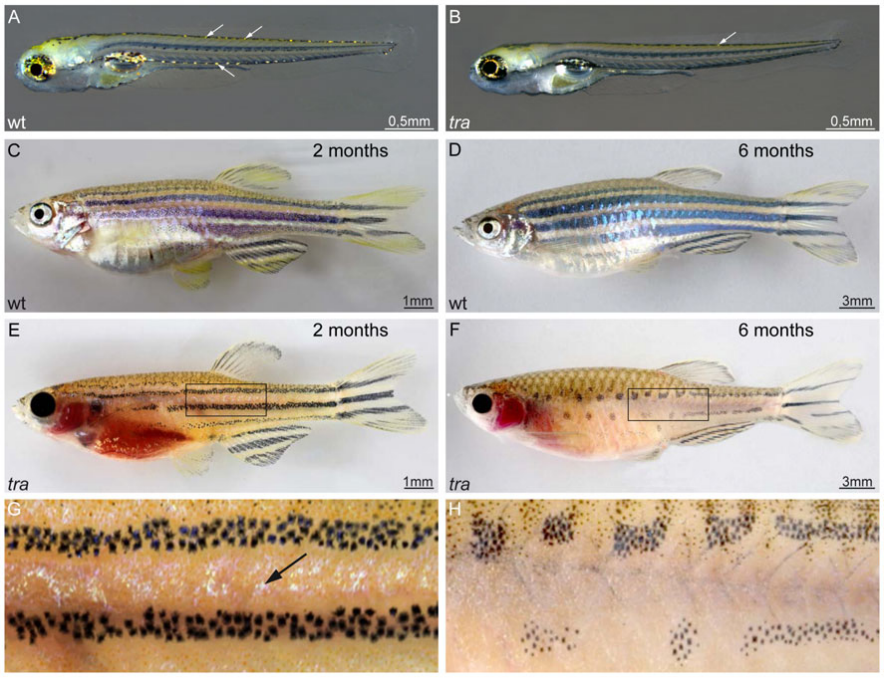
*transparent* mutants show a reduction in iridophore pigmentation. (**A**,**C**,**D**) Wild type and (**B**,**E–H**) *tra* mutants. At 5 dpf, mutant larvae show strong reduction in iridophores in dorsal and ventral positions (B) as compared to wild type (A). Arrows in panels A and B highlight the appearance and position of iridophores in larvae. No other defects are apparent. Two months old wild type animals (C) present a fully developed pigment pattern, while *tra* individuals (E) developed only few iridophores, visible between the dark stripes. (G) Close-up of the region boxed in panel E. At that age, melanophores are reduced in number in the mutants, however, the typical four stripes developed. While the mutant fish grow, the melanophore stripes break up into spots (F, close up in H), compare six months old *tra* mutant (F) to wild type (D) of the same age. The abdominal cavity, typically covered by a thick sheet of iridophores in wild type, lacks this cell type in *tra* mutants. Iridophores of the eyes are strongly reduced throughout life in the mutants. Pigment patterning of fins and scales appear normal. Scale bars: 0.5 mm (A,B); 1 mm (C,E), 3 mm (D,F).

*transparent (tra)* mutants exhibit a strong reduction of iridophore pigmentation throughout development (Fig. 1). Larvae at 5 dpf show strongly reduced iridophore pigmentation in the dorsal and ventral trunk and tail positions (Fig. 1B, compare to wild type in Fig. 1A). The most striking reduction in iridophore pigmentation can be observed in the yolk sac stripe, which develops between 4 and 5 dpf (not shown). Melanophores distribute in normal numbers and positions in *tra* mutant larvae. There are no obvious defects in the distribution and appearance of xanthophores. The reduction in iridophore pigmentation in later stages of development occurs gradually. During metamorphosis, the first interstripe, close to the horizontal myoseptum, forms almost normally, however, soon thereafter, the silvery pigmentation is gradually lost (Fig. 1E,F, compare to wild type in Fig. 1D,C). Young *tra* adults show some iridophore pigmentation, especially in the first interstripe (Fig. 1E,G). Besides the reduction in iridophore pigmentation, *tra* mutant adults also show a remarkable reduction of melanophores (Fig. 1F,H). Overall, the phenotype in larvae and adults is similar to the phenotype of *shd/ltk* albeit less severe. However, in contrast to *shd*, iridophore pigmentation is also strongly reduced in the eyes of *tra* mutants at all stages of development. As in other adult viable iridophore mutants like *rse*, *shd* and *bonaparte* (Lang et al., 2009; Lopes et al., 2008; Parichy et al., 2000a), the reduction of melanophores affects only the trunk while caudal and anal fins are normally striped. *tra* mutants show no other morphological defects and are adult viable and fertile.

### *tra* mutants display defects in iridophore maintenance

**Fig. 2.**
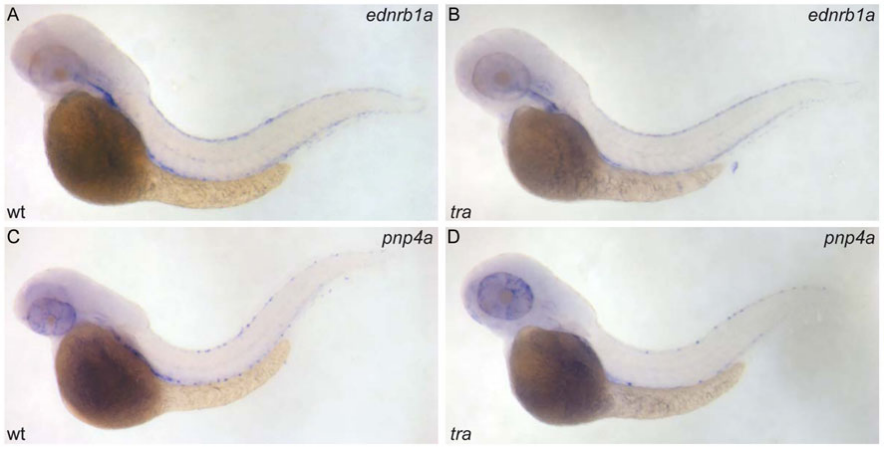
*transparent* affects pigment synthesis of iridophores, but not their specification and initial differentiation. *ednrb1a* RNA hybridization of wild type (**A**) and *tra* mutant larva (**B**), *pnp4a* RNA hybridization of wild type (**C**) and *tra* mutant (**D**); 48 hpf.

First, we wanted to find out if the reduction in silvery pigment is due to a defect in iridophore specification, differentiation or maintenance. We performed RNA *in situ* hybridization on zebrafish embryos for *ednrb1* and *pnp4a* transcripts, which both are expressed in iridoblasts (Lang et al., 2009; Lister et al., 2006). At 2 dpf the number of cells labelled for either of the two probes in dorsal trunk positions in *tra* embryos was normal, compared to wild type (Fig. 2). However, during subsequent stages, the number of cells expressing *pnp4a* was reduced. At 5 dpf, only very few (0–5) *pnp4a* positive iridophores are present in the dorsal trunk of *tra* larvae compared to 20–30 in wild type (20 larvae each genotype were analysed, data not shown). During adult development the decrease in iridophore pigmentation can be directly observed. In young adults silvery pigmentation is still visible in the first and dorsal interstripe regions, albeit reduced, but it is completely lost during further maturation. We conclude that iridophore specification and initial differentiation occur normally in *tra* mutants, but it appears that these cells are lost subsequently.

To test if the loss of iridophores is caused by apoptosis, we used TUNEL and acridine orange labelling during larval development to detect dying cells. We found no difference between wild type and *tra* mutants in both assays (data not shown). Either the loss of iridophores is not caused by apoptosis or the number of dying cells is too low at any one specific time-point to be detected by our methods. As shown below, we detect a small number of dying iridophores in the retina, while most iridophores are lost. There is the remote possibility that iridophores in the body are still present, but gradually fail to produce pigment as well as the ability to interact with melanophores in stripe formation (see below).

### *transparent* acts cell-autonomously in iridophores

**Fig. 3.**
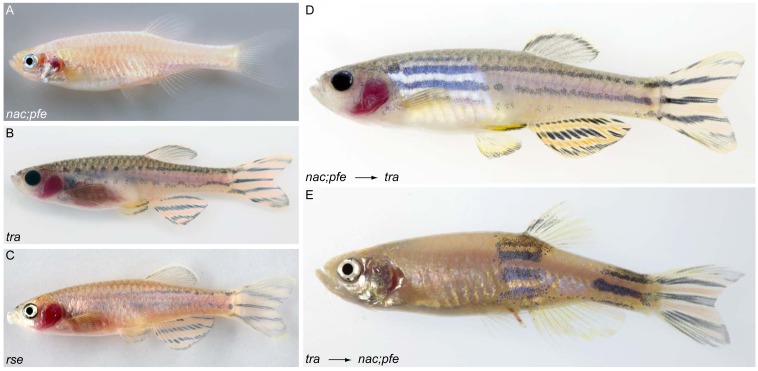
*transparent* is required cell-autonomously in iridophores. (**A–C**) Mutants used in transplantation experiments: (A) *nac;pfe*, (B) *tra*, (C) *rse*. (**D**) *nac;pfe* mutant which received *tra* mutant xanthophores and melanophores, showing that both cell types contribute normally to stripes. (**E**) *tra* mutant which received *nac;pfe* mutant iridophores, showing that the supply with iridophores is sufficient to normalize the pigment pattern.

As both melanophores and iridophores are affected in *tra* mutant adults, we created chimeric animals to address in which cell type *tra* is required. At first, we introduced wild type iridophores into mutants by transplanting cells at the blastula stage. As donors, we used *nacre;pfeffer (nac;pfe)* double mutants. In *nac/mitfA* mutants, melanophores do not develop while xanthophore development in *pfe/fms* is inhibited caused by the absence of Fms signaling (Lister et al., 1999; Parichy et al., 2000b). Double mutant *nac;pfe* therefore develop iridophores as the only chromatophore type, which cover broad regions of the adult flank (Fig. 3A). We transplanted *nac;pfe* donor blastula cells into *tra* mutant host blastula stage embryos and raised the resulting chimeras to adulthood. If *tra* was required only in iridophores, we expected to find a local display of donor-derived iridophore pigmentation in the adult flank. As shown in Fig. 3E adult chimeras developed big regions (“clones”) on their flanks with completely normal appearing iridophores in the interstripes (compare to tra adult Fig. 3B). This result indicates that *tra* is required in iridophores. For confirmation that the transplantation of normal iridophore precursor cells is causing this rescue, we transplanted *rse* mutant donor cells into *tra* blastulas. It has been shown, that *rse* acts cell-autonomously in iridophores (Frohnhöfer et al., 2013) (*rse* adult in Fig. 3C). In this experiment, we did not find any chimera with clones of restored iridophore pigmentation (0/78) compared to 20% (8/40) in the *nac;pfe* into *tra* transplantations, indicating that *rse* and *tra* are affected in the same cell type.

Strikingly, in the clones resulting from the transplantation of *nac;pfe* embryos into *tra* mutant hosts a full restoration of the pigment cell arrangement of the melanophore stripes is observed (Fig. 3E). They do not only show restored iridophore pigmentation but also a normal display of melanophore stripes. As the donor embryos do not provide melanophore precursors, these melanophores must be of the *tra* mutant genotype. To see, if *tra* mutant melanophores are indeed functional in stripe formation, we performed the reciprocal transplantation and transplanted cells from *tra* mutant donor blastulas into *nac;pfe* mutant hosts. In this experiment, the donor provides *tra* melanophores to a recipient that lacks melanophores but has normal iridophores. Again, the adult chimeras showed large patches of restored wild type-like pigment patterning (14/105, Fig. 3D).

Taken together, we conclude, that the *tra* gene product is required cell-autonomously in iridophores, as iridophore pigmentation is restored by *nac;pfe* but not *rse* mutant cells, and that *tra* mutant melanophores and xanthophores are fully functional. In consequence, this shows that the deficit in melanophores visible in *tra* mutant adults is a result of the strong iridophore reduction. This in turn indicates that during normal development iridophores interact with melanophores and thus actively participate in adult pigment pattern formation.

### *transparent* encodes the mitochondrial protein Mpv17

**Fig. 4.**
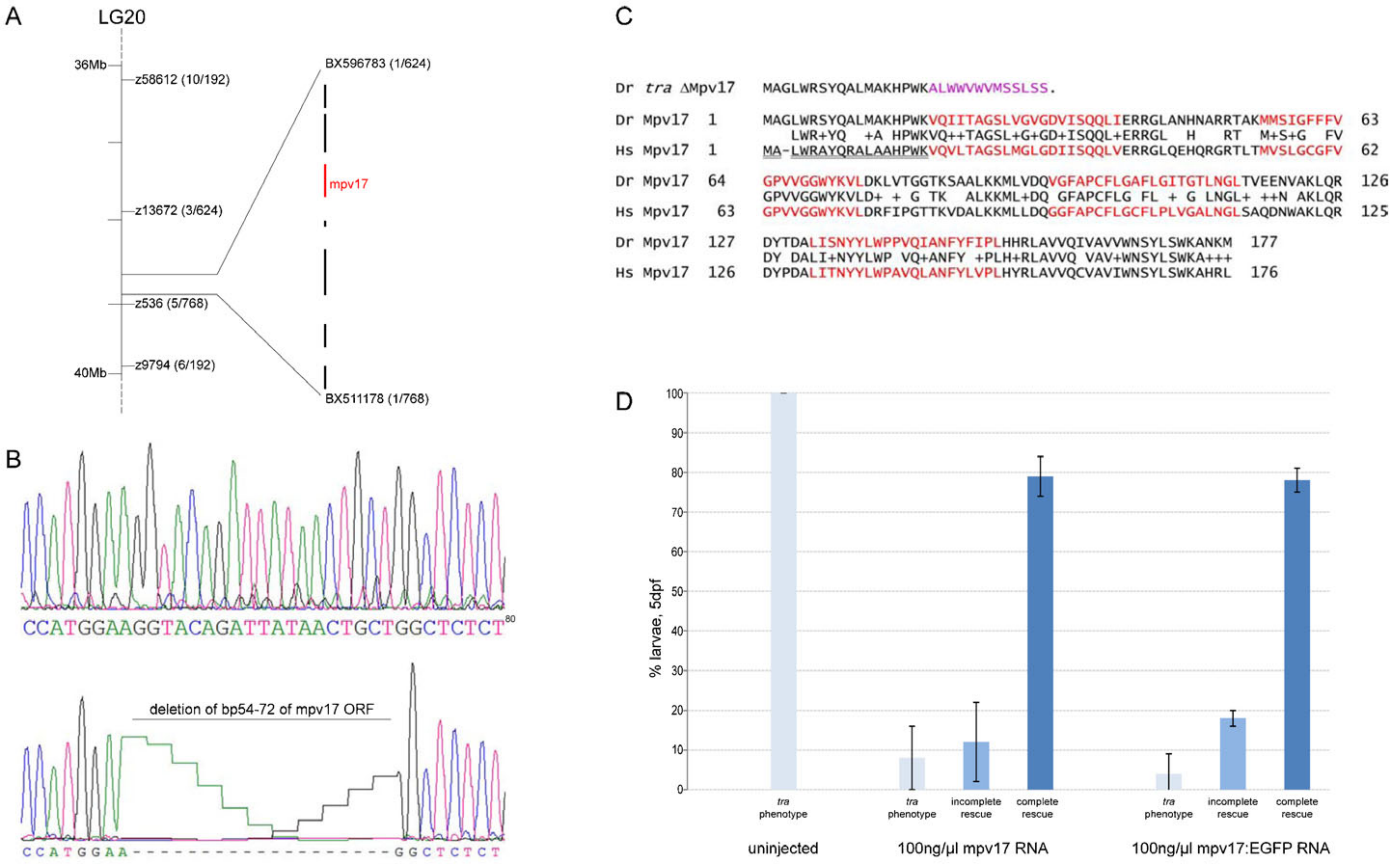
*transparent* encodes Mpv17. (**A**) *tra* maps to chromosome 20. The genomic interval was mapped by the indicated microsatellites (z-marker) and further refined by SNPs located on contigs BX596783 and BX511178. Brackets give the number of recombinants in relation to the total number of mutant individuals. In this closest interval, 7 genes are annoted. (**B**) Sequencing revealed a deletion in the *mpv17* coding sequence in *tra* mutants, resulting in a frameshift (D, amino acids in pink) and an early stop codon. (**C**) Comparison of zebrafish (Dr) Mpv17 protein sequence and its human homolog (Hs). The proteins show 69% amino acid identity. Amino acids forming the transmembrane domains are highlighted in red. The mitochondrial target signal in the human protein sequence is underlined. (**D**) Injection of mpv17 or mpv17:egfp encoding mRNA into *tra* mutants rescue larval iridophore pigmentation (n(uninjected) = 167, n(mpv17) = 299, n(mpv17:egfp) = 300).

To identify the gene affected in the *tra* mutant, we mapped the mutation to an interval on linkage group 20 flanked by the markers z13672 and z536. Further fine mapping using single nucleotide polymorphisms located *tra* within a 200 kb genomic interval between 38.6 and 38.8 Mb (Fig. 4A). We sequenced most of the annotated genes in the interval and identified a splicing defect in the mRNA encoding Mpv17. This results in a small deletion in the mature mRNA producing a frame shift following codon 18 and a premature stop after 31 codons (Fig. 4B,C). The aberrant mRNA seems to be caused by the use of a cryptic splice donor site in exon 2, thus deleting the remaining part of this exon, and subsequent normal fusion with exon 3. In contrast to wild type, a 2 kb genomic region covering the predicted splice site of exon 2 could not be amplified from *tra* mutant larvae. Therefore, the deletion most likely includes bigger parts of intron 2 (data not shown).

We probed for the spatial and temporal expression of *mpv17* by RNA *in situ* hybridization in early development. Using two independent RNA probes we found that *mpv17* is ubiquitously expressed at low levels throughout embryos and larvae (data not shown). We did not detect any transcript enrichment in a neural crest specific pattern or in iridophores.

To test if the identified deletion in *mpv17* is causative for the *tra* mutant phenotype, we injected one-cell stage mutant embryos with *in vitro* transcribed mpv17 mRNA at a concentration of 100 ng/µl. Approximately, 80% of these injected embryos developed visibly pigmented iridophores in normal numbers and were phenotypically indistinguishable from wild type at 4 dpf (Fig. 4E). We conclude that the deletion in the *mpv17* gene leads to the *tra* phenotype. We further raised the injected embryos to adulthood. All injected animals developed the typical *tra* mutant phenotypes, indicating that with the RNA supplement, only the larval phenotype could be rescued (data not shown). This indicates that the *mpv17* gene product is required in iridophores throughout development.

Blast searches for other Mpv17 proteins encoded in the zebrafish genome revealed the presence of four genes encoding proteins with considerable similarities. The highest similarity was found for Mpv17-like2, with 52/158 identical amino acids and 87/158 similar amino acids. Two more Mpv17-like proteins (Mpv17l1a, Mpv17l1b) are significantly less similar, with approximately 25% identical and 40% similar residues. Pmp22, which serves as the founding member of this protein family, shows 27% identical and 44% similar residues. Mammalian Mpv17 proteins contain N-terminal mitochondrial localization signals and are located in the inner mitochondrial membrane *in vivo* (Spinazzola et al., 2006). The same is true for Sym1p, the Mpv17 ortholog of yeast. Human Mpv17 was shown to be able to substitute for Sym1p, indicating a highly conserved function of this protein (Trott and Morano, 2004). Therefore, we used targetp v1.1 and MitoprotII v1.101 to test for a potential mitochondrial localization of zebrafish Mpv17 and its orthologs. Mpv17, Mpv17-like 2 and Mpv17-like 1a are predicted to localize to mitochondria with a probability of more than 0.8, whereas for Mpv17-like1b a mitochondrial localization seems to be less likely. However, no cleavable N-terminal signal peptide was predicted for zebrafish Mpv17, leaving it open if the zebrafish protein is indeed targeted to mitochondria.

### Zebrafish Mpv17 localizes to mitochondria

**Fig. 5.**
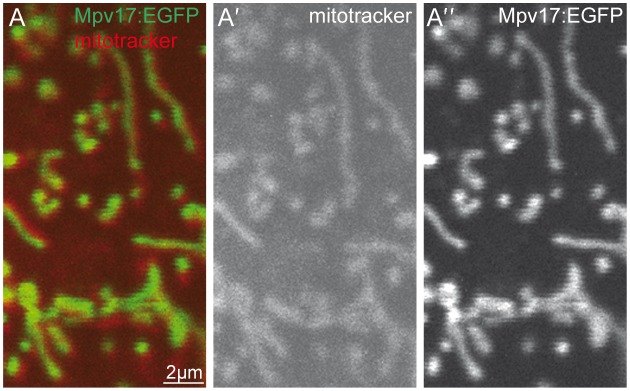
Zebrafish Mpv17 colocalizes with mitochondria. (**A**) Mpv17 C-terminally tagged with EGFP (green, **A″**) colocalizes with mitochondria labelled by mitotracker (red, **A′**). Image was taken from larval epidermal cell at 4 dpf. Scale bar: 2 µm.

To test if Mpv17 localizes to mitochondria, we expressed Mpv17 C-terminally tagged with GFP by injection of the respective mRNA into one-cell stage embryos. Injections into *tra* mutants showed rescue ability comparable to its untagged version, confirming the functionality of the fusion variant (Fig. 4E). The subcellular localization of the fusion protein was analyzed by staining of the resulting larvae with mitotracker followed by confocal microscopy. Labelling analysis revealed a colocalization of Mpv17 with mitochondria in diverse cell types like epidermal cells, muscle cells and hair cells of lateral line neuromasts (Fig. 5). From these data we conclude that zebrafish Mpv17 localizes to mitochondria, in agreement with the subcellular distribution reported for its yeast and mammalian homologs. However, the distribution of mitochondria and Mpv17 could not be observed in iridophores, as labeling signals were quenched due to the strong reflective properties of these cells. Our attempt to analyse the subcellular localization of Mpv17:EGFP fusion protein in iridophores by electron microscopy was not successful, leaving it open, if Mpv17 is restricted to mitochondria in iridophores as well.

**Fig. 6.**
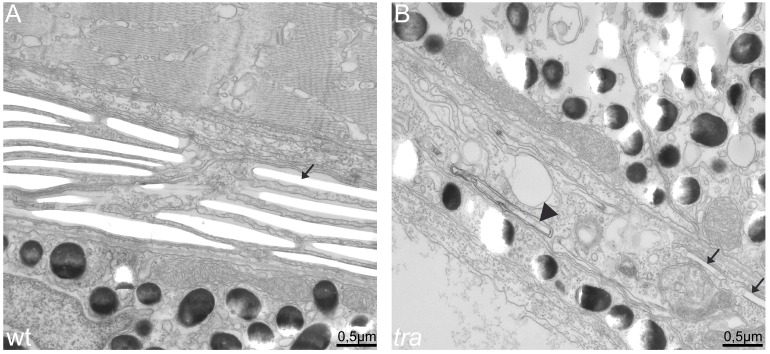
*transparent* mutant iridophores display characteristics of apoptotic cells. EM sections of wild type (**A**) and *transparent* mutant iridophores (**B**) in the eye of 5 dpf larvae. (A) Wild type iridophores develop stacks of iridosomes nearly completely filling the cells. Iridosomes are not contrasted and appear empty as the guanine crystals are lost during the sectioning process (arrow). (B) Iridosomes are also visible in *tra* mutants. In the cell shown, only two iridosomes seem to contain guanine crystals (arrows), whereas others contain contrastable material (arrowhead), indicating that guanine deposition is not completed. Importantly and in contrast to wild type, *tra* mutant iridophores contain many vesicles, a sign for apoptosis. Scale bars: 0.5 µm.

By electron microscopy, we analyzed the phenotype of *tra* iridophores in greater detail. Due to the rather scarce number of iridophores in the trunk which renders their identification in thin sections very difficult, we chose to compare eye iridophores of larvae of wild type, *tra* mutants and *tra* mutants injected with *mpv17* mRNA. In wild type, iridophores can be easily identified based on the regular distribution of iridosomes, which appear as membrane-encased flat organelles often organized in stacks (arrow in Fig. 6A). The lumen of iridosomes appears whitish, as the reflective crystalline material is lost during sectioning (Fig. 6A). As expected, the number of clearly identifiable iridophores containing at least a few iridosomes is dramatically reduced in *tra* mutants (Fig. 6B). Typically, only few of those cells could be found in eye cross-sections of mutant larvae, whereas in comparable sections of wild type, iridophores form a nearly continuous layer. In agreement with the phenotype described above, *tra* mutant iridophores develop a few rudimentary iridosomes (arrows in Fig. 6B). However, these cells accumulate high amounts of vesicles that often contain multiple membranes and seem to be filled with cellular contents, features characteristic for autophagosomes. In contrast to this observation, fully developed wild type iridophores are nearly completely occupied by crystal-filled iridosomes and do not contain such vesicles. These observations support the notion that in *tra* iridophores degenerate. Injection of *mpv17* and *mpv17:EGFP* encoding mRNAs into *tra* mutant embryos lead to the development of iridophores in the retina which are numerically and morphologically indistinguishable from wild type (data not shown).

## Discussion

Our study provides evidence that the *transparent* mutant phenotype is caused by a deletion in the *mpv17* gene causing a frameshift in the open reading frame and thus resulting in an early truncation of the protein. We found that Mpv17 protein localizes to mitochondria in several different cell types of zebrafish larvae, as was shown for its homologs in mammals and yeast (Spinazzola et al., 2006; Trott and Morano, 2004). Strikingly, mutations in *mpv17* in mice and humans lead to severe phenotypes resulting in early lethality of the affected individuals, which contrasts with the viability of the zebrafish *tra*/*mpv17* mutants. Mice carrying an insertion in the *mpv17* gene abolishing protein function develop mitochondrial DNA depletion, most prominently in the liver, and late onset glomerulosclerosis, hair graying and neurodegeneration of the peripheral nervous system (Meyer zum Gottesberge et al., 2012; Müller et al., 1997; Viscomi et al., 2009; Weiher et al., 1990). Likewise, several mutations in *mpv17* were reported to be associated with mitochondrial DNA depletion syndrome in humans causing death during early childhood (AlSaman et al., 2012; El-Hattab et al., 2010; Navarro-Sastre et al., 2010; Spinazzola et al., 2008; Spinazzola et al., 2006; Wong et al., 2007). In contrast to mammalian *mpv17* mutants, we did not find depletion in mitochondrial DNA content of liver, muscle and brain tissue isolated from *tra* mutant adult animals (data not shown). This is in agreement with the very specific iridophore phenotype of the *tra* mutants and their good viability. A possible explanation for this might be redundancy, and that paralogs of *mpv17* carry out the vital functions of this gene in other cells. This seems likely as there are more Mpv-like proteins encoded in the zebrafish genome than in mammals.

Given the ubiquitous expression of *mpv17* in vertebrates, it seems reasonable to speculate, that the loss of Mpv17 function might result in tissue-specific phenotypes only under special cellular circumstances, like aging, stress or special metabolic requirements. In zebrafish, enzymes involved in guanosine metabolism are highly expressed in iridophores at stages when *pnp4a* transcripts accumulate, and their knockdown causes loss of iridophores in larvae not only in the trunk but also in the eyes (Ng et al., 2009). These phenotypes are reminiscent of the larval *tra* phenotype. Iridophores accumulate high amounts of guanine deposited as crystals in iridosomes. Because of the high deposition of guanine in its crystalized, solid form in iridophores, the equlibrium of the reaction catalyzed by Pnp4a should be strongly shifted towards guanine. This results in a continuous withdrawal of guanine precursors, such as guanosine and its nucleosides and nucleotides, so that, dGTP, required for mitochondrial DNA replication, might become limiting, therefore requiring active import of either its precursors or dGTP itself. Interestingly, in humans the loss-of-function of GUOK, a mitochondrial matrix enzyme involved in guanosine nucleotide salvage pathway, also causes mitochondrial DNA depletion syndrome with a comparable phenotypic outcome to *mpv17* mutations (Mandel et al., 2001).

In another scenario, Mpv17 would be directly involved in iridosome biogenesis by possibly localizing to iridosome membranes, ensuring somehow the correct deposition of guanine crystals. By electron microscopy, we found iridosomes in the few iridophores left in *tra* mutants. However, these cells were filled with membrane-bound vesicles reminiscent to autophagosomes, which we could not find in wild type iridophores indicating that these cells are apoptosing. However, the fact that *tra* mutant iridophores are able to develop iridosomes and guanine crystals at least to some degree, favours the idea of a mitochondrial defect with secondary loss of iridophores by cell death.

Interestingly, *shd* and *tra* show very similar reductions in melanophores, despite the fact that *shd* affects the specification of iridophore precursors whereas *tra* leads to the death of differentiated iridophores. From transplantation experiments (this study) (Frohnhöfer et al., 2013), we conclude that melanophores accumulate in sufficient numbers to form a continuous stripe only in the presence of differentiated iridophores forming a dense interstripe. In this context it is interesting to note that the diverse pigment cell types seem to differentiate in a sequential way during metamorphosis, with iridophores being the first cell type to differentiate, then, soon afterwards, xanthophores appear. Both cell types form the first interstripe, which is almost complete when the first melanophores start to accumulate in dark stripes dorsally and ventrally. Following this, new interstripes with iridophores and xanthophores develop dorsally and ventrally to the dark stripes. This sequence of events offers the possibility that iridophores supply a so far unknown factor required for melanophore development and maintenance.
